# State minimum wage increases delay marriage and reduce divorce among low‐wage households

**DOI:** 10.1111/jomf.12832

**Published:** 2022-03-11

**Authors:** Benjamin R. Karney, Jeffrey B. Wenger, Melanie A. Zaber, Thomas N. Bradbury

**Affiliations:** ^1^ Department of Psychology, Pritzker Hall University of California, Los Angeles Los Angeles California USA; ^2^ RAND Corporation Santa Monica California USA

**Keywords:** divorce, low‐income, marriage, minimum wage, public policy

## Abstract

**Objective:**

To estimate the effects of state‐level changes in the minimum wage on marriage and divorce among low‐wage earners.

**Background:**

Proponents of raising the minimum wage highlight the potential benefits of increased earnings for low‐income families, yet to date research on the effects of raising the minimum wage has focused almost exclusively on economic outcomes. No research has yet documented whether these changes actually affect marriage and divorce.

**Method:**

Using the Current Population Survey and the American Community Survey, this project applied a quasi‐experimental difference‐in‐difference method to exploit similarities between states that have, and have not, raised their minimum wage.

**Results:**

Across data sources, among men and women earning low wages, a one‐dollar increase in the state minimum wage predicts a 3%–6% decline in marriage entry and a 7%–15% decline in divorce one and 2 years later.

**Conclusion:**

Both changes are likely to strengthen low‐income families, and are substantially larger effects than those obtained by federal policies directly targeting interpersonal dynamics within low‐income couples.

**Implications:**

Government policies that reduce stress on couples and facilitate their access to resources may improve family outcomes, invisibly and without making additional demands on the time of couples who are already strained.

**Abbreviations**
ACS: American Community Survey
CPS: Current Population Survey
FPL: Federal Poverty Line
GDP: Gross Domestic Product
OLS: Ordinary Least Squares
SES: Socioeconomic Status

## INTRODUCTION

Although individuals with low incomes aspire to marriage and stable partnerships as much as more affluent individuals (Trail & Karney, 2012), their aspirations are less likely to be fulfilled. Compared to those with higher SES, poorer individuals are less likely to marry and five times more likely to have children out of wedlock (Lundberg & Pollak, 2015). When they do marry, they marry earlier (Lundberg et al., 2016) and are roughly twice as likely to divorce (Raley & Sweeney, 2020). Couples with low incomes, therefore, have less access to the benefits associated with satisfying and enduring marriages, including greater emotional (Whitton et al., 2014), financial (Zissimopoulos et al., 2013), and physical well‐being (Robles et al., 2014), and better academic, social, and psychological outcomes for their children (Brown, 2010).

Recognizing the benefits of healthy partnerships and the disproportionate challenges faced by low‐income couples, federal policymakers over the past two decades have allocated nearly a billion dollars toward education programs designed to promote better communication skills and stronger relationships among this vulnerable population (Heath, 2012). Yet large‐scale multisite evaluations of these programs indicate that they have been ineffective (Lundquist et al., 2014; Manning et al., 2014; Wood et al., 2014), leading some to suggest that directly targeting the external stressors that challenge low‐income communities could prove a viable alternative direction for efforts to support couples and families within these communities (Karney et al., 2018).

In view of theory (Conger & Conger, 2008) and evidence (Burstein, 2007; Schneider, 2015) indicating that financial security promotes family well‐being, one way of pursuing this suggestion is to evaluate the effects on families of policies designed to improve the economic circumstances of low‐income households (Holt‐Lunstad et al., 2017). To the extent that widening income inequality has been identified as an explanation for the divergent experiences of higher‐ and lower‐income families (Carbone & Cahn, 2014), social policies that strengthen the financial security of individuals living with low‐incomes should therefore also affect their decisions to enter and terminate committed partnerships. Indeed, federal and local policies that are not explicitly designed to promote family outcomes nevertheless appear to do so. For example, the Affordable Care Act, by giving young adults the option of obtaining health insurance without relying on a partner, lowered rates of marriage and cohabitation, while increasing rates of divorce (Abramowitz, 2015). In contrast, spouses who receive health insurance through their partners are less likely to divorce than spouses with their own sources of health insurance (Sohn, 2015).

If policy makers were to pursue an agenda aimed at promoting economic well‐being among low‐income families, what policies might they target? One noteworthy policy explicitly designed to achieve this goal is raising the minimum wage (Smith, 2015). Although the federal minimum wage was established in 1935, it has only rarely been raised to match inflation. In response to federal inaction, state and municipal governments began to raise their minimum wages above the federal threshold in the 1990s and 2000s. Some of these increases have been quite large by historical standards. For example, in 2007, Ohio increased the state‐level minimum wage from $5.15 to $6.85/h (a 33% increase), thereby raising a full‐time, full‐year minimum wage worker's wage from $10,300 per year to $13,700—enough to move a single‐earner family of two above the 2007 federal poverty level. In 2016, the state of California moved to raise the minimum wage in all counties, affecting 5.6 million people (Jacobs & Perry, 2016), and in 2019, raises took effect in 21 additional states (Kiersz, 2018). Current proposals to increase the federal minimum wage to $15 would the affect more than 27 million workers, over a quarter of whom are parents and over a third of whom are married (Congressional Budget Office, 2019).

To date, analyses of the impact of minimum wage increases have focused primarily on individual and household economic outcomes (Congressional Budget Office, 2014). In line with this focus, debates about the merits of raising the minimum wage revolve around whether or not employers affected by these increases subsequently hire fewer workers, potentially *reducing* incomes among low‐wage earners (Machin & Manning, 1996). Evidence for such effects has so far been mixed. Some reviewers conclude that “moderate increases in the minimum wage are a useful means of raising wages in the lower part of the wage distribution that has little or no effect on employment and hours” (Belman & Wolfson, 2014, p. 397). Consistent with this view, raising the minimum wages of food service workers across six cities had no negative effects on employment but did lead to significant increases in earnings (Allegretto et al., 2018). A more recent review, examining the entire set of published studies in the “new minimum wage” literature since 1992, concluded that 78.9 percent of the 69 published studies (121 estimates) reported a negative employment effect (Neumark & Shirley, 2021, p. 3). However, those effects tended to be small—in less than half of the studies with negative effects did the authors' preferred estimate reach statistical significance at the *α* = 0.05 level.

While economists appear to be converging on the view that minimum wage increases yield small and, in most cases, non‐statistically significant negative effects on employment, evidence is clearer that wage increases do increase earnings reliably for most minimum wage earners. Indeed, proponents of raising the minimum wage refer explicitly to the potential of higher wages to promote more stable families when justifying their positions (Hall & Cooper, 2012). Thus, it is reasonable to expect that legislation designed to increase wages should also affect the formation and dissolution of committed partnerships in lower‐income communities. Yet, despite consistent appeals to family well‐being, and despite the changes in the minimum wage in localities across the country over the last two decades, no one has yet documented whether changes in the minimum wage in fact affect family outcomes like marriage and divorce.

In the absence of direct evidence, the impacts of raising the minimum wage on family outcomes like marriage and divorce remain an open question. To the extent that financial strain represents a salient challenge within lower‐income communities, family stress theories (Patterson, 2002) suggest that policies that enhance financial stability should strengthen intimate relationships and thereby facilitate transitions into marriage within those communities. Forty‐five years of data from the Panel Study of Income Dynamics support this view, revealing that declines in men's income and employment help to account for declines in rates of first marriage over that time (Schneider et al., 2018). From this perspective, policies that increase the earnings of low‐income individuals should reduce barriers to marriage and thereby predict higher rates of entry into marriage.

Financial considerations also play a substantial role in whether couples consider their relationships worth maintaining. Economic stress and financial strain do in fact predict less satisfying and stable marriages (Jackson et al., 2017; Williams et al., 2013), and higher levels of poverty and consumer debt predict greater vulnerability to divorce (Copen et al., 2012). To the extent that increased earnings reduce the financial strain experienced by low‐income couples, couples in communities that have raised the minimum wage should experience more satisfying relationships and thereby lower rates of divorce.

Yet, despite the consistency of research informed by family stress perspectives, Life History Theory (Giudice et al., 2015) makes strikingly different predictions. The Life History approach posits that people adopt different reproductive strategies across their lifespan depending on the resources available to them and the consequent adaptiveness of different choices. This perspective suggests that, rather than speeding entry into marriage, higher earnings may delay marriage among low‐income individuals, as individuals who are less resource‐constrained can spend more time searching for an appropriate partner. Consistent with this perspective, low‐income women who win modest jackpots are not more but rather less likely to marry, compared to matched peers (Hankins & Hoekstra, 2011). If raising the minimum wage increases the earnings of low‐income individuals, they may delay entry into marriage, bringing their marital timing closer in line with more affluent individuals. To the extent that later marriages are less likely to dissolve than earlier marriages (Rotz, 2016), then these effects would ultimately lead to more stable marriages, consistent with predictions guided by family stress theory.

To date, we are aware of no prior research that addresses these alternative perspectives, and more generally no research that extends research on the effects of raising the minimum wage to acknowledge their potential effects on family outcomes. The proposed research fills these gaps by evaluating whether raising the minimum wage affects marriage and divorce among low‐wage individuals. To address these questions, we implemented a quasi‐experimental approach to investigate policy effects by exploiting similarities between states in the US that did or did not change their minimum wage. This design has several advantages over other methods of evaluating these changes. First, multiple years of data allow us to estimate and difference out a state‐specific time trend *before* the first minimum wage change takes effect, removing baseline variability between states (e.g., a cultural predisposition to different family formation patterns; differences in average wages) and ensuring parallel trends before the first wage change. Second, multiple changes per state allow us to estimate a separate state‐specific time trend *after* the first minimum wage change (accounting for, e.g., shifts in the age of first marriage, as well as trends in labor markets unrelated to the wage change that might impact family formation). Finally, this method lends itself to straightforward estimators that are robust and easy to evaluate. Since the now‐classic Card and Krueger (1994) analysis of the New Jersey increase in the minimum wage, researchers have exploited geographic differences in minimum wages; here we apply this method for the first time to marriage and divorce as outcomes.

## METHODS

### Data sources

Our analyses used data covering reference years 2004 through 2015 from two independent monthly surveys that differ in their scope, format, and aims, although both are approximately representative of US households at the state level. The Current Population Survey (CPS) is a primarily telephone‐based survey of approximately 60,000 households in populous areas, designed to be representative of the labor force; the American Community Survey (ACS) is a primarily mail‐based survey of approximately 300,000 households in all geographic areas, designed to be representative of the broader population. As the analyses focused on family formations among potential minimum wage‐earning adults, only individuals aged 18–35, who comprise the majority of minimum wage earners (U. S. Bureau of Labor Statistics, 2018), were included (descriptive statistics for the samples are presented in Table 1).

**TABLE 1:** Descriptive statistics, ACS and CPS sample 2003–2014

	CPS men	CPS women	ACS men	ACS women
Population of 18‐ to 35‐year‐olds (*N*)	263,539	282,575	3,959,347	3,957,870
In households <$20/h	49.1%	51.4%	51.3%	53.9%
In households <$16/h	38.2%	40.6%	41.5%	43.5%
In households <200% FPL	26.8%	36.9%	38.0%	42.4%
Underlying sample (*N*)	132,866	148,229	1,908,715	2,060,308
Age	26.4	26.5	26.3	26.5
Ever married	34.9%	44.6%	35.0%	43.9%
Divorced	3.1%	4.5%	3.5%	5.0%
State‐year panel (*N*)	612	612	612	612
Ever married	42.9%	50.8%	41.0%	48.8%
Divorced	3.4%	5.1%	3.4%	5.1%

*Note*: Authors' analysis of ACS and CPS. Main analysis covers reference years 2004 to 2015. Statistics weighted using survey‐provided individual‐level weights; note that CPS weights are designed to be representative of the civilian labor force, whereas ACS weights are designed to be representative of the civilian population.

Abbreviations: ACS, American Community Survey; CPS, Current Population Survey; FPL, Federal poverty line.

### Operationalizations

**FIGURE 1:**
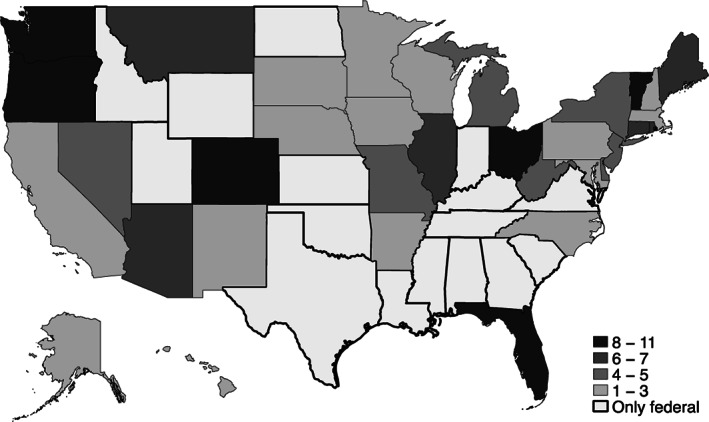
Number of state‐level minimum wage increases 2002–2015

We transformed each survey data set into a state‐level panel by taking the mean of key variables (e.g., currently divorced, ever married, household/family earnings, hours worked) by state, year, and gender, using survey‐provided individual weights to ensure state and national representativeness. For example, we calculated the fraction of males ages 18 to 35 who had ever been married in Ohio in 2005, and treated that as one observation in the resulting aggregate panel. Each regression specification had a sample size of 51 states and districts providing data annually over 11 years. As we did not expect immediate translation of minimum wage increases, we explored the impacts of policy changes on marriage and divorce at 1‐ and 2‐year lags. We did not examine further lags to avoid any chance that estimates of the effects of one adjustment to the minimum wage are affected by a second adjustment within the same state (see Figure 1).

By aggregating the data to a state‐year analysis, we used state‐level variation in minimum wage policies to compare outcomes between states where a minimum wage had increased and control states that had not raised the minimum wage. We excluded municipal wage changes in our analysis, likely biasing our estimates toward zero: there may have been additional “doses” of treatment lumped in with control observations or with smaller state increases.

We did not classify individuals' wage status directly by wages earned for two reasons. First, the known heaping in wage reporting (i.e., the tendency for respondents to round their reports of income to the nearest round number) would have threatened our ability to identify precisely who is affected by minimum wage changes. Second, indirect classification avoids introducing the endogeneity of being a minimum wage earner—a minimum wage increase may encourage entry into the labor force, into employment, and into minimum wage employment specifically. Instead, following prior work (Campolieti et al., 2005), we defined low‐wage earners by factors likely to expose individuals or households to minimum wage impacts. Because marriage and divorce are likely household‐level decisions influenced by shared resources, we applied the classification of low‐wage to all members of the household (in the case of multiple adults): if at least one adult in a two‐adult household met our criteria for low‐wage, both adults received this classification. We recognize that this may include some two‐earner households with higher combined earnings that may not be traditionally seen as low‐wage. Through the labor force participation of the lower‐wage earner, the household is still potentially sensitive to economic changes affecting low‐wage families, and so we use the term “low‐wage” for brevity. In our preferred specification, individuals were defined to be low wage if their individual hourly earnings were less than or equal to 20 dollars an hour. To evaluate the robustness of our results, we also explored two alternate definitions: one with a stricter earnings threshold (16 dollars an hour) and one based on household earnings relative to the federally‐defined poverty level.

### Analysis strategy

Identification of a causal effect relies on the between‐state, over‐time variation in state minimum wages. Looking within a single state with a minimum wage change would grant over‐time variation, but any estimates would be local to that state (with low out‐of‐sample validity), and would suffer from any endogeneity related to the timing of the minimum wage change. Pooling all of the states together across many years allows each state to serve as its own control, permitting state‐level adjustment for parallel trends in addition to a posttreatment time trend. Thus, we used a quasi‐experimental difference‐in‐difference methodology, estimating both the difference within‐state (over time) and the difference between states that differ in their timing of minimum wage increases.

### Econometric specification

Our state‐level approach rests on two key assumptions. The first is that trends in control states and in states changing their minimum wages would have followed parallel trajectories in the absence of a minimum wage increase. To confirm that this assumption was satisfied in our model, we detrended data for each state‐gender panel, using only observations *before* each state's first observed minimum wage change. We also controlled for the logarithm of state GDP and recessionary periods to account for other time‐varying economic impacts on marriage and divorce.

The second assumption is that effects in the post‐change period can be attributed only to the minimum wage change. An external factor affecting the number of marriages or divorces in a state while or shortly after a minimum wage change occurred would bias our estimates by incidentally absorbing that impact. We thus estimated a second trend during the period after the first minimum wage change, which was feasible because nearly all states with a minimum wage change in our analysis frame had enacted multiple changes between 2003 and 2015 (see Figure 1 and Table S1).

We estimated the effect of states' minimum wage premia—defined as the amount that each state's minimum wage exceeded the federal minimum wage ‐ on the detrended state proportion of the low‐wage population married (or divorced, in separate analyses) denoted by $y_{\mathrm{st}}^{d}$. We include controls for changes in the federal minimum wage level and indicators for recession periods denoted as $\tau_{t};$ and two sets of time‐varying state‐level controls: state GDP and a linear state‐specific pretreatment time trend indicated by $\hat{\phi_{\mathrm{st}}}$. Using the population defined as “low wage,” we modeled marriage (and separately, divorce) rates as:

(1) $$y_{\mathrm{st}}^{d}=\beta_{0}+\beta_{1}\mathrm{pre}m_{\mathrm{st}}+\hat{\phi_{\mathrm{st}}}+\tau_{t}+\epsilon \;$$

where $\phi_{\mathrm{st}}$ is the predicted outcome of interest in a state‐specific, gender‐specific regression with a linear time trend and no other controls, estimated using only the pretreatment observations for each state. $\mathrm{pre}m_{\mathrm{st}}$ is the amount by which a given state's minimum wage exceeds the federal minimum wage in a given year. Thus, a state with a state minimum wage equal to the federal minimum wage has a premium of zero.

The use of pretreatment and posttreatment state‐specific time trends permit us to interpret minimum wage premia as minimum wage changes while controlling for the state context that may have led to the change. Thus, $\beta_{1}$ gives the average change in family formation (e.g., a state's proportion of 18‐ to 35‐year‐old men who have ever married in year t) in response to a one‐dollar increase in the state minimum wage *in excess* of any federal requirements. We estimate each model in the CPS and ACS separately to evaluate whether our results replicate within each independent dataset.

## RESULTS

### Effects on earnings and hours worked

For state‐level minimum wage increases to benefit low‐wage couples, the policy must raise household earnings within this population. Yet some have argued that, as labor becomes more expensive (due to the statutory rise in minimum wages), firms may hire fewer workers or cut hours, thereby reducing household earnings. To evaluate this possibility, we used our same difference‐in‐difference approach to examine wages within low‐wage households likely to be sensitive to minimum wage changes, defined as those in which at least one worker was earning $20 per hour or less. Our estimates found no evidence for a negative effect of minimum wage increases on earnings among this population. In fact, state‐level minimum wage increases had significant *positive* effects on earnings and no effect on hours worked for at‐risk men and women both 1 and 2 years after the minimum wage increase (see Tables S2 and S3). Because state minimum wage increases did increase household earnings as expected, we turned to estimating their effect on marriage and divorce.

### Effects on rates of marriage

Table 2 presents ordinary least squares estimates of the effect of minimum wage increases (measured as a premium, or dollars above the federal minimum) on marriage rates from 2003 to 2015. As the table reveals, a $1 per hour increase in the state minimum wage (in excess of the federal) reduced rates of marriage by approximately two percentage points, implying that low‐wage earning individuals are foregoing marriage, at least temporarily, when their state minimum wage is increased. The effect size was similar across data sets (although slightly larger in the ACS data), similar for men and women, and consistent in the year that the minimum wage increase was enacted and in the following year. Expressed in terms of the percentage reduction in marriages as a result of increasing minimum wages, this translates to a 5% reduction in men's marriage rates 1 year after passage of the minimum wage increase, and a 5% to 6% reduction 2 years after passage. Corresponding marriage rate reductions for women are 4.5% and 3% to 5%.

**TABLE 2:** Effects of state minimum wage on marriage

Minimum wage	Current Population Survey		American Community Survey	
	Males 18–35	Females 18–35	Males 18–35	Females 18–35
1‐year lag	−0.020	−0.023	−0.021	−0.021
	(0.006)	(0.006)	(0.005)	(0.006)
2‐year lag	−0.022	−0.017	−0.024	−0.026
	(0.005)	(0.006)	(0.005)	(0.006)

*Note*: Coefficient estimates and (standard errors) from eight separate OLS regressions (2× survey, 2× gender, 2× lag); we reject the null hypothesis of no minimum wage effect at the *α* = .01 level in each regression. Additional controls include: recession indicator, federal minimum wage level, logarithm of state gross domestic product, a linear time trend (with intercept) prior to the adoption of the state minimum wage, and a separate post‐adoption time trend. Population: Households with at least one worker earning $20 per hour or less. Dependent variable: proportion of state population ever married.

### Effects on rates of divorce

Table 3 presents estimates of the effect of minimum wage increases on divorce. Overall, we found that increases in state minimum wages were associated with lower rates of divorce in both datasets for men and in ACS data for women. The results from the CPS data for women were still negative, but did not reach significance due to the smaller sample size. Once again, we found similar effect sizes across the data sets with the ACS generally showing slightly larger effects. In all cases, differences across the data sets were not significant (see Table S7). Expressed in terms of the percentage reduction in divorce as a result of increasing minimum wages, we found that a $1 per hour increase in the minimum wage resulted in a 10% to 15% reduction in divorce rates for men and an 7% to 12% reduction for women at 1‐ and 2‐year lags. These are substantively large effects, consistent with the effects of the Minnesota Family Investment Program, which reduced divorce rates by 25% among participants (Gennetian, 2003), and much larger than the Supporting Healthy Marriage study which, despite spending over $11,000 per couple, found no significant impact on whether couples stayed married at the 12‐ and 30‐month follow‐ups (Lundquist et al., 2014).

**TABLE 3:** Effects of state minimum wage on divorce

Minimum wage	Current Population Survey		American Community Survey	
	Males 18–35	Females 18–35	Males 18–35	Females 18–35
1‐year lag	−0.0052	−0.0006	−0.0035	−0.0040
	(0.0017)	(0.0019)	(0.0011)	(0.0013)
2‐year lag	−0.0033	0.0034	−0.0035	−0.0049
	(0.0018)	(0.0031)	(0.0011)	(0.0014)

*Note*: See notes from Table 2. We reject the null hypothesis of no minimum wage effect at the α = .01 level in each ACS regression. Within the CPS, we reject the null hypothesis for males at a 1‐year lag at the α = .01 level, and for males at a 2‐year lag the α = .05 level. For CPS females, we fail to reject the null hypothesis of no effect at conventional levels of α. Controls and population definition are the same as Table 2. Dependent variable: proportion of study population currently divorced and not remarried.

### Robustness

To evaluate the robustness of our results, we conducted several additional tests. First, we estimated a series of placebo treatment regressions where states were randomly assigned the minimum wage history of another state, and then re‐estimated our baseline regressions. We conducted this test 1000 times and found that the minimum wage effect was never statistically correlated with marriage or divorce outcomes in these randomization tests (see Figure S1). This effectively rules out the likelihood that increases in the minimum wage are spuriously correlated with other state‐wide outcomes that are driving marriage and divorce rates.

We also explored different low‐wage definitions (e.g., household being below 200% of the federal poverty line) and a specification which pooled men and women together. All of these results (see Tables S4–S6) provide added support that state‐level minimum wage increases were related to reductions in marriage and divorce among those 18–35 and in low‐income households. In sum, we find robust effects on family formation that are consistent across different definitions of the low‐wage population, different data sets (ACS and CPS), and model specification. Notably, we observed effects of state‐level policy changes on low‐wage workers even though we did not restrict our analyses to minimum wage earners specifically. It is likely that we would have observed stronger results with analyses that could identify workers directly affected by minimum wage increases, but this would require panel data that tracked individuals over time in order to capture effects on multiple jobholding, labor market entry, and job switching.

## DISCUSSION

To date, debates over the effects of raising the minimum wage have focused narrowly on their economic impact (Cengiz et al., 2019), overlooking their potential effects on family outcomes. Expanding the scope of this discussion is long overdue, as a number of states and large cities have recently initiated increases in their local minimum wages and there is political momentum to increase the federal minimum wage as well (Kiersz, 2018). By comparing states that did institute a change to states that did not, while holding constant stable and time‐varying differences between states, the current analyses are the first to estimate the effects of state‐level minimum wage increases on marriage and divorce among low‐wage households.

A preliminary result of this analysis is to confirm that, when states increase their minimum wage, incomes among low‐wage households do in fact rise significantly without increasing hours worked. Even as debates about the minimum wage continue to invoke concerns about the potential for disemployment and thus income reductions as a consequence of higher labor costs (e.g., Machin & Manning, 1996), these findings join a growing literature that fails to find strong evidence for such effects (Cengiz et al., 2019; Dube et al., 2010).

Commensurate with these effects, we find robust evidence that state‐level increases in the minimum wage predict lower rates of marriage and divorce 1 and 2 years following such changes. Although it remains possible that rising incomes spurred marriages for low‐wage earners who were already coupled, the net decline in marriage among unmarried individuals supports Life History perspectives (Giudice et al., 2015), which suggest that when low‐wage earners find their jobs more remunerative, they have the resources to extend their search for appropriate long‐term mates. Among the younger population we studied (18–35 years old), this is likely to represent a delay in marriage, rather than a decision to forgo marriage entirely. In this way, raising the minimum wage appears to bring the marital timing of low‐wage earners more in line with the timing of more affluent individuals, who also tend to marry at older ages (Lundberg et al., 2016).

Because later marriages are on average less likely to end in divorce (Rotz, 2016), a consequence of this effect should be more stable marriages among low‐wage earners in the long term, which may explain some of the reductions in divorce observed in these analyses. In the short term, the observed reductions in divorce may be better explained by Family Stress theories, which suggest that higher incomes made it easier for lower wage‐earning couples to adapt to stress and maintain their romantic connections.

Estimated effects on marriage and divorce were strikingly similar across the CPS and the ACS, despite the differences in scope and methods of the two surveys. Nevertheless, several factors constrain interpretation of these results. First, although we compared results across 1‐ and 2‐year lags, other effects may emerge at longer lags. These analyses could not determine whether declines in rates of marriage and divorce represented transitions deferred or decisions to forgo these transitions entirely. Second, our analyses focused on the younger workers who represent over half of minimum wage earners (U. S. Bureau of Labor Statistics, 2018); weaker effects might be observed among older workers (e.g., above age 35), whose marriages are likely to be more established. Third, while our strategy isolates the impact of state minimum wage changes, we cannot determine with certainty whether our observed effects are driven by an increase in take‐home earnings, a change in labor hours, a change in confidence about oneself or the future, or some other mechanism.

In sum, during the same years that federal policymakers spent nearly a billion dollars on educational programs that had negligible effects on marriage and divorce, states that simply raised their minimum wages delayed marriages and reduced divorce rates significantly among low‐wage earners. Future efforts to strengthen low‐income families should therefore explore additional policy levers, such as expanding the earned income tax credit (Michelmore, 2018) or access to health care (Sohn, 2015), that may have additional benefits to family formation and stability. As other scholars have argued (Johnson, 2012; Williamson et al., 2017), government policies that reduce stress on couples and facilitate their access to resources may improve family outcomes, invisibly and without making additional demands on the time of couples who are already strained. Understanding the specific factors that mediate the association between these policies and relationship decision‐making could strengthen future efforts to assist families living with low incomes.

## CONFLICT OF INTEREST

The authors declare that they have no conflict of interest.

## Supporting information


**Appendix S1**. Supporting Information

## References

[jomf12832-bib-0001] Abramowitz, J. (2015). Saying, “I Don't”: The effect of the affordable care act young adult provision on marriage. Journal of Human Resources, 51(4), 933–960. 10.3368/jhr.51.4.0914-6643R2

[jomf12832-bib-0002] Allegretto, S. A. , Godoey, A. , Nadler, C. , & Reich, M. (2018). The new wave of local minimum wage policies: evidence from six cities. Center on Wage and Employment Dynamics, Institute for research on Labor and Employment, University of California, Berkeley.

[jomf12832-bib-0004] Belman, D. , & Wolfson, P. J. (2014). What does the minimum wage do?. W.E. Upjohn Institute for Employment Research. 10.17848/9780880994583

[jomf12832-bib-0005] Brown, S. L. (2010). Marriage and child well‐being: Research and policy perspectives. Journal of Marriage and Family, 72(5), 1059–1077. 10.1111/j.1741-3737.2010.00750.x 21566730 PMC3091824

[jomf12832-bib-0006] Burstein, N. R. (2007). Economic influences on marriage and divorce. Journal of Policy Analysis and Management, 26(2), 387–429. 10.1002/pam.20257

[jomf12832-bib-0007] Campolieti, M. , Fang, T. , & Gunderson, M. (2005). Minimum wage impacts on youth employment transitions, 1993–1999. Canadian Journal of Economics/Revue canadienne d'économique, 38(1), 81–104. 10.1111/j.0008‐4085.2005.00270.x

[jomf12832-bib-0008] Carbone, J. , & Cahn, N. (2014). Marriage markets: How inequality is reshaping the American family. Oxford Press.

[jomf12832-bib-0009] Card, D. , & Krueger, A. B. (1994). Minimum wages and employment: A case study of the fast food industry in New Jersey and Pennsylvania. The American Economic Review, 84(4), 772–793.

[jomf12832-bib-0010] Cengiz, D. , Dube, A. , Lindner, A. , & Zipperer, B. (2019). The effect of minimum wages on low‐wage jobs. The Quarterly Journal of Economics, 134(3), 1405–1454. 10.1093/qje/qjz014

[jomf12832-bib-0011] Conger, R. D. , & Conger, K. J. (2008). Understanding the processes through which economic hardship influences families and children. In D. R. Crane & T. B. Heaton (Eds.), Handbook of families and poverty (pp. 64–81). Sage Publications.

[jomf12832-bib-0012] Congressional Budget Office . (2014). The effects of a minimum‐wage increase on employment and family income. Congress of the United States, Congressional Budget Office.

[jomf12832-bib-0052] Congressional Budget Office. (2019). The Effects on Employment and Family Income of Increasing the Federal Minimum Wage. Congress of the United States.

[jomf12832-bib-0014] Copen, C. E. , Daniels, K. , Vespa, J. , & Mosher, W. D. (2012). First marriages in the United States: Data from the 2006–2010 National Survey of family growth. In National health statistics reports; 49. National Center for Health Statistics. 22803221

[jomf12832-bib-0015] Dube, A. , Lester, T. W. , & Reich, M. (2010). Minimum wage effects across state borders: Estimates using contiguous counties. The Review of Economics and Statistics, 92(4), 945–964.

[jomf12832-bib-0016] Gennetian, L. A. (2003). The long‐term effects of the Minnesota family investment program on marriage and divorce among two‐parent families. https://www.mdrc.org/sites/default/files/full_567.pdf

[jomf12832-bib-0017] Giudice, M. D. , Gangestad, S. W. , & Kaplan, H. S. (2015). Life history theory and evolutionary psychology. In D. M. Buss (Ed.), The handbook of evolutionary psychology. John Wiley & Sons, Inc. 10.1002/9781119125563.evpsych102

[jomf12832-bib-0019] Hall, D. , & Cooper, D. (2012). How raising the federal minimum wage would help working families and give the economy a boost. Economic Policy Institute.

[jomf12832-bib-0020] Hankins, S. , & Hoekstra, M. (2011). Lucky in life, unlucky in love? The effect of random income shocks on marriage and divorce. Journal of Human Resources, 46, 403–426.

[jomf12832-bib-0021] Heath, M. (2012). One campaign under God: The campaign to promote marriage in America. New York University Press.

[jomf12832-bib-0022] Holt‐Lunstad, J. , Robles, T. F. , & Sbarra, D. A. (2017). Advancing social connection as a public health priority in the United States. American Psychologist, 72(6), 517–530. 10.1037/amp0000103 28880099 PMC5598785

[jomf12832-bib-0023] Jackson, G. L. , Krull, J. L. , Bradbury, T. N. , & Karney, B. R. (2017). Household income and trajectories of marital satisfaction in early marriage. Journal of Marriage and Family, 79(3), 690–704. 10.1111/jomf.12394 28603296 PMC5464617

[jomf12832-bib-0024] Jacobs, K. , & Perry, I. (2016). $15 minimum wage in California: who would be affected by the proposal to raise California's minimum wage?. UC Berkeley Labor Center.

[jomf12832-bib-0025] Johnson, M. D. (2012). Healthy marriage initiatives: On the need for empiricism in policy implementation. American Psychologist, 67, 296–308. 10.1037/a0027743 22468785

[jomf12832-bib-0026] Karney, B. R. , Bradbury, T. N. , & Lavner, J. A. (2018). Supporting healthy relationships in low‐income couples: Lessons learned and policy implications. Policy Insights From the Behavioral and Brain Sciences, 5(1), 33–39. 10.1177/2372732217747890

[jomf12832-bib-0027] Kiersz, A. (2018). The minimum wage is set to increase in 21 states and DC in 2019 — here's what it will be in every state. *Business Insider*. Retrieved December 29, 2018, from https://www.businessinsider.com/minimum-wage-2019-state-map-2018-12

[jomf12832-bib-0028] Lundberg, S. , & Pollak, R. A. (2015). The evolving role of marriage: 1950‐2010. The Future of Children, 25(2), 29–50. http://www.jstor.org/stable/43581971

[jomf12832-bib-0029] Lundberg, S. , Pollak, R. A. , & Stearns, J. (2016). Family inequality: Diverging patterns in marriage, cohabitation, and childbearing. Journal of Economic Perspectives, 30, 79–102. 27170828 10.1257/jep.30.2.79 PMC4861075

[jomf12832-bib-0030] Lundquist, E. , Hsueh, J. , Lowenstein, A. E. , Faucetta, K. , Gubits, D. , Michalopoulos, C. , & Knox, V. (2014). *A family‐strengthening program for low‐income families: Final impacts from the Supporting Healthy Marriage Evaluation* (Vol. OPRE Report 2014‐09A). Office of Planning, Research and Evaluation, Administration for Children and Families, U.S. Department of Health and Human Services.

[jomf12832-bib-0031] Machin, S. , & Manning, A. (1996). Employment and the introduction of a minimum wage in Britain. The Economic Journal, 106, 667–676.

[jomf12832-bib-0032] Manning, W. D. , Brown, S. L. , Payne, K. K. , & Wu, H. S. (2014). Healthy marriage initiative spending and U.S. marriage & divorce rates, a state‐level analysis (FP‐14‐02) .

[jomf12832-bib-0033] Michelmore, K. (2018). The earned income tax credit and union formation: The impact of expected spouse earnings. Review of Economics of the Household, 16, 377–406. 10.1007/s11150-016-9348-7

[jomf12832-bib-0034] Neumark, D. , & Shirley, P. (2021). Myth or measurement: What does the new minimum wage research say about minimum wages and job loss in the United States?. (Working Paper 28388). National Bureau of Economic Research. http://www.nber.org/papers/w28388

[jomf12832-bib-0035] Patterson, J. (2002). Integrative family resilience and family stress theory. Journal of Marriage and Family, 64(2), 349–360.

[jomf12832-bib-0036] Raley, R. K. , & Sweeney, M. M. (2020). Divorce, Repartnering, and stepfamilies: A decade in review. Journal of Marriage and Family, 82(1), 81–99. 10.1111/jomf.12651 PMC10817771 38283127

[jomf12832-bib-0037] Robles, T. F. , Slatcher, R. B. , Trombello, J. M. , & McGinn, M. M. (2014). Marital quality and health: A meta‐analytic review. Psychological Bulletin, 140(1), 140–187. 10.1037/a0031859 23527470 PMC3872512

[jomf12832-bib-0038] Rotz, D. (2016). Why have divorce rates fallen?: The role of women's age at marriage. Journal of Human Resources, 51(4), 961–1002.

[jomf12832-bib-0039] Schneider, D. (2015). Lessons learned from non‐marriage experiments. Future of Children, 25, 155–178.30679897

[jomf12832-bib-0040] Schneider, D. , Harknett, K. , & Stimpson, M. (2018). What explains the decline in first marriage in the United States? Evidence from the panel study of income dynamics, 1969 to 2013. Journal of Marriage and Family, 80, 791–811. 10.1111/jomf.12481

[jomf12832-bib-0041] Smith, L. (2015). Reforming the minimum wage: Toward a psychological perspective. American Psychologist, 70(6), 557–565. 10.1037/a0039579 26348337

[jomf12832-bib-0042] Sohn, H. (2015). Health insurance and risk of divorce: Does having your own insurance matter? Journal of Marriage and Family, 77(4), 982–995. 10.1111/jomf.12195 26949269 PMC4772968

[jomf12832-bib-0043] Trail, T. E. , & Karney, B. R. (2012). What's (not) wrong with low‐income marriages? Journal of Marriage and Family, 74, 413–427. 10.1111/j.1741-3737.2012.00977.x

[jomf12832-bib-0044] U. S. Bureau of Labor Statistics . (2018). *Characteristics of minimum wage workers, 2017* (BLS Reports, Issue 1072). https://www.bls.gov/opub/reports/minimum-wage/2017/pdf/home.pdf

[jomf12832-bib-0046] Whitton, S. W. , Rhoades, G. K. , & Whisman, M. A. (2014). Fluctuation in relationship quality over time and individual well‐being: Main, mediated, and moderated effects. Personality and Social Psychology Bulletin, 40, 858–871. 10.1177/0146167214528988 24727811 PMC5790156

[jomf12832-bib-0047] Williams, D. T. , Cheadle, J. E. , & Goosby, B. J. (2013). Hard times and heart break: Linking economic hardship and relationship distress. Journal of Family Issues, 36(7), 924–950. 10.1177/0192513x13501666 PMC4470497 26097273

[jomf12832-bib-0048] Williamson, H. C. , Karney, B. R. , & Bradbury, T. N. (2017). Education and job‐based interventions for unmarried couples living with low incomes: Benefit or burden? Journal of Consulting and Clinical Psychology, 85(1), 5–12. 10.1037/ccp0000156 27775415

[jomf12832-bib-0050] Wood, R. G. , Moore, Q. , Clarkwest, A. , & Killewald, A. (2014). The long‐term effects of building strong families: A program for unmarried parents. Journal of Marriage and Family, 76(2), 446–463. 10.1111/jomf.12094

[jomf12832-bib-0051] Zissimopoulos, J. M. , Karney, B. R. , & Rauer, A. J. (2013). Marriage and economic well being at older ages. Review of Economics of the Household, 1‐35, 1–35. 10.1007/s11150-013-9205-x

