# Supplementary material for: State minimum wage increases delay marriage and reduce divorce among low‐wage households
Source: J Marriage Fam. 2022 Mar 11;84(4):1196–207. doi: 10.1111/jomf.12832 (PMC9545266; doi:10.1111/jomf.12832)
Supplement: Supplementary file 1 — Appendix S1. Supporting Information [file JOMF-84-1196-s001.docx]

Supplementary Materials for:

State Minimum Wage Increases Delay Marriage and Reduce Divorce

Among Low-Wage Households

Benjamin R. Karney, Jeffrey B. Wenger, Melanie A. Zaber, Thomas N. Bradbury

Correspondence to:  [karney@psych.ucla.edu](mailto:xxxxx@xxxx.xxx)

**This PDF file includes:**

Materials and Methods

Supplementary Text

Tables S1 to S7

Fig. S1

Materials and Methods

Data summary and descriptive statistics

*The Current Population Survey (CPS)*: The CPS is a monthly, household-based survey conducted by the US Census Bureau for the US Department of Labor. Each month approximately 60,000 households are surveyed. Interviews are typically conducted in person with subsequent interviews conducted via telephone using a computer-assisted telephone interview (CATI). One individual within each household is the survey respondent (typically either the leaseholder or owner). The respondent answers questions about all members of the household over the age of 15 and provides household composition information about those 15 and under. Because the CPS has a complicated address-based stratified sampling scheme, we have used design weights throughout the analysis and for creating state-level averages. To hold down costs, the survey uses a 4-8-4 rotation group structure: the same households are sampled for four months in a row, they then leave the survey for 8 months, and then return to the survey for another four months. Households in the outgoing rotation groups are asked about their labor-market earnings.

The primary purpose of the CPS is to determine national and state-level unemployment rates. The CPS Annual Social and Economic Supplement is also the official source of national poverty measures. Consequently, the survey focuses considerable attention on the labor market activities and earnings of the members of the households. Included in the labor data are industry and occupation. Additionally, demographic data are collected about age, race, sex, marital status and education level. Finally, information about the structure of the household and all of the relationship statuses are available, offering a way to distinguish between different families within a household.

*The American Community Survey (ACS)*: The ACS is a household-based survey administered completely by mail, reaching approximately 300,000 U.S. households each month. It is compulsory in that respondent completion of the survey is required by law. As in the CPS, one person fills out the full survey for all members of the household. Similar to the CPS, the ACS instructions request that the householder (person who is renting or owns the residence) is the respondent. However, the ACS is different from the CPS in key ways. Although the ACS is also fielded monthly (across different individuals), responses are aggregated into a yearly sample. The ACS is focused on population representation rather than labor force representation, and also has a broader geographic sample (the ACS includes all counties in each state, unlike the CPS).

The ACS does not contain measures of hourly earnings or exact hours worked in a year. During our analysis frame^[[1]](#footnote-1)^, the ACS asks weeks worked per year in an interval format. The ACS asks “How many weeks DID this person work, even for a few hours, including paid vacation, paid sick leave, and military service?” with response choices of 50 to 52 weeks, 48 to 49 weeks, 40 to 47 weeks, 27 to 39 weeks, 14 to 26 weeks, and 13 weeks or less. Following the UC Berkeley Labor Center (2015), we set the number of weeks worked to the midpoint of the interval reported, and then calculate hourly wages by dividing earnings by the product of the weeks midpoint and usual hours worked per week.

Although the data in both surveys are collected at the household level, we use the data recorded at the individual level using the appropriate person weights. This allows us to perform state aggregations of the proportion of people married or divorced in a particular year.

*Measures – Marriage and Divorce*: The CPS asks the following question to determine the marital status of each member of the household. “(Are / Is) (name/you) now married, widowed, divorced, separated or never married?” Respondents can answer in six ways 1) married - spouse PRESENT. 2) married - Spouse ABSENT, 3) widowed, 4) divorced, 5) separated, 6) never married.

The ACS asks questions about the current marital status of each person in the household. The question is worded “What is this person’s marital status?” and there are four possible answers: widowed, divorced, separated, never married.

For both surveys, we classify an individual as ever married if they respond anything other than “never married,” and we classify an individual as divorced only if they report “divorced.”

Descriptive statistics for each sample are available in Table S1. Note that the ACS sample is an order of magnitude larger; however, descriptive statistics between the two samples are quite similar with the exception of the proportion below 200 percent of the federal poverty line and the fraction divorced. Once aggregated to the state-year panel, the differences in divorce between samples disappear.

Supplementary Text

Minimum Wage Policy

The minimum wage was enacted in 1938 under the Fair Labor Standards Act; since that time the Federal government has raised the wage 22 times. The original minimum wage was set at $.25 per hour. The 1980s ushered in a long period of no increases of minimum wages. In 1980, the minimum wage was set at $3.35 and remained at that level until 1990. From 1997 to 2007 the minimum wage remained at $5.15/hour. The current minimum wage ($7.25/hour) has not been increased since July 24, 2009. Since 1980, there have been three decade-long periods of minimum wage stasis. Starting in the early 1980s, once state legislatures and governors realized that a Federal minimum wage increase was unlikely, many states started raising their minimum wages. As of 2019, 30 states had minimum wages set above the Federal threshold. There have been a large number of state-level changes in minimum wages. According to Cengiz, Dube, Lindo, and Zentler-Munro (2019), there have been 172 “prominent” minimum wage changes from 1979-2019.

Data compiled by David Neumark provides all the state-level minimum wages from 1960-2019.^[[2]](#footnote-2)^ We cross checked these data against a similar database produced by Kavya Vaghul and Ben Zipperer.^[[3]](#footnote-3)^ In cases where the data did not agree (on either statutory minimum wage, or timing of enactment), we returned to the original state-level legislation and coded accordingly.

Table S1 shows the size of the minimum wage premium across all states and for those with positive premia. Many states have a zero minimum wage premium since they do not have a state-level minimum wage. We note that these results are not population-weighted. We treat each state as a separate experiment and therefore discuss the state-level aggregate results. On average, the 50 states (and Washington, D.C.) have a minimum wage premium of 38 cents. However, for those states with a positive premium the value increases to 96 cents; consequently, the difference between states with and without a state-level minimum wage is approximately one dollar at any given time. The average difference within a given state between one year and the next is 22 cents; however, this includes states that make no change in a given year. Limiting to states that experience a change (driven by Federal or state changes), the average difference is 54 cents, but limiting to states that experience a state-driven change yields a slightly smaller difference of 50 cents. Finally, the average state experiences about five and half minimum wage changes over the panel (some of which are incremental changes due to inflation-indexing of the state minimum wage), but recall that there are 3 federal wage changes in our analysis period. Removing those means that the average state experiences approximately 3 state-driven minimum wage changes. Note that federal minimum wage changes do not necessitate minimum wage changes in all states—if a state’s established minimum wage already exceeds the new federal minimum wage, no change occurs.

One caveat is that some statutory increases in minimum wage occurred at the sub-state level, including cities such as Los Angeles, San Francisco, Seattle and Albuquerque among others. These sub-state increases may have further influenced marriage and divorce outcomes in ways not captured in our analyses. However, given the design of this analysis – using state-level changes in minimum wages in different states over different time periods resulting in geographic and implementation rate variation across a wide range of states – it is implausible that city-specific effects are driving statewide changes.

Minimum Wage Effects on Earnings and Hours Worked

The mechanism that we postulate is that higher incomes increase the resources available to low-wage earners; consequently, they are more likely to delay marriage entry and, if already married, less likely to exit marriage via divorce. In order for these hypotheses to be valid, we need to ensure that the minimum wage does in fact raise household incomes without adding additional burdens to couples by motivating more hours spent at work. This may seem obvious, but if minimum wage increases result in widespread job loss (as economic theory suggests may be the case in some markets), wages could fall on average.

Table S2 demonstrates that is not the case. For all of the groups analyzed across both data sets, the minimum wage raised household (CPS) and family (ACS) incomes. Household income, as used in the CPS, is a slightly broader measure of income and consequently we see a higher rate of increase as compared to the ACS. As expected, results attenuate over time with the two-year lagged results being somewhat lower than the same- or one-year results. Effect sizes are in line with the size of the minimum wage increases discussed earlier. For states with a minimum wage increase the average increase is $.50 or about $1,000 per year for a full-time, full-year worker. This translates to about 7 percent on a $14,000 per year income which, in turn, is about the gross earnings of a minimum wage worker at the federal minimum wage of $7.25 per hour. It is possible that the increase in earnings stems not from more remunerative hours, but rather from simply working *more* hours. Table S3 demonstrates that is not the case either. For all of the groups analyzed across both data sets, no significant increases in hours were detected. The only even marginally significant results suggests a decrease in hours worked per week (18- to 35-year old men in the two-year lag specifications).

Estimates Using Different Analysis Samples

In Table S4, we present results for different analysis samples. We show the less than $20 per hour results as a baseline (replicating the results in the main text). We then define a second analysis population to be adult members of a household earning less than 200 percent of the federal poverty level, a narrow definition in terms of total population as shown in Table 1 in the main text. Our third analysis population are households where one worker earns less than $16 per hour. Focusing on the ACS results where sample sizes are largest and estimates are most precise, we find results that are similar across all the populations. The 200 percent of federal poverty level sample and $16 per hour sample results are quite similar to the $20 per hour sample. The $16 per hour sample has slightly smaller estimates but they are all negative and statistically significant. The estimates for divorce are similarly robust in the ACS data.

The CPS data provide similarly robust estimate for marriage and divorce; all point estimates are negative and at least four of six estimates for each marriage and divorce are statistically significant at conventional levels ($\alpha=0.05$). Note, however, that none of our tests of statistical significance control for joint hypothesis testing. In this table alone there are 24 results, random chance would suggest that one of them would fail to be statistically significant ($\alpha=0.05$). Overall, we find no evidence that our choice of low-wage definition is driving our results.

Model and Point Estimate Robustness

In this section we discuss the sensitivity of our results based on the model specification. In Table S5 we present results for men and women who are ages 18 to 35 together. We return to our baseline population (those living in a household where at least one adult earns less than $20 per hour). Once again, we find robust parameter estimates for both the CPS and ACS data for both marriage and divorce outcomes. We fail to achieve statistical significance for the CPS, two-year lagged divorce outcome, but the parameter estimate is negative and substantively similar. Again, we note that the CPS data, owing to a smaller underlying sample size, is likely to have larger standard errors.

Table S6 contains estimates of 24 separate OLS regressions using the ACS data with the population consisting of those in a household with a worker earning less than $20 per hour. The estimates are the coefficients on the minimum wage premium variable in a one-year lag specification. The control variables are progressively increasing with the exception of columns (5) and (6) which consist of two different time trend specifications.

These estimates show remarkable consistency across specifications. All coefficient estimates are negative across all specifications. The effect of the minimum wage on divorce for women is not statistically significant in the absence of time controls, but a correct specification requires them (econometrically these are not trend-stationary, so eliminating the time control leads to misspecification). Overall, the results in Tables S5 and S6 show that the parameter estimates are stable across multiple specifications and control variables.

We also conducted a similar specification test with the CPS data (not shown, but available upon request) with broadly similar results—robust consistency in marriage, good consistency in divorce.

Other specification issues

The econometric literature has highlighted several challenges with using a “difference-in-differences” approach with different implementation timings (as this paper does). First, Wolfers (2006) notes the need to distinguish between stock and flow impacts. Policies can affect the baseline taste for marriage or divorce, or the barriers/incentives to marriage and divorce, and the interaction of these affects what is observed in both the number of unions/dissolutions (stock) and the rates (flow). Because Wolfers studies a policy—unilateral divorce—that is adopted only once by a given state (in contrast to minimum wage changes, implemented multiple times by adopting states), he is able to estimate both the short-term impacts and the new long-run equilibrium.

Second, Goodman-Bacon (2018) highlights the challenges raised by differential implementation timing. In “classic” difference-in-differences estimation, policy take-up is determined at a single point, clearly delineating the pre- and post-treatment periods for control and treatment states. With different windows for implementation that vary by state, there is no true “pre-treatment” period for a control, leading pre-treatment but eventually-treated states to be used as controls. The resulting comparisons are weighted by their respective variances, and the treatment effect biased if treatment effects vary over time (especially important if we think there is selection into the timing of adoption).

In follow-on work, Baker et al. (2021) show that staggered timing does not bias estimates in the case of constant and equal treatment effects. But with constant and unequal effects, or more problematically, with dynamic effects, the estimates are biased. These concerns are mitigated somewhat by the number of never-treated states, which limits the number of pre-treatment “treated” states used as controls.

Note that this literature focuses on policies that take on a binary form (implemented or not implemented), whereas this analysis uses a continuous measure (minimum wage *premium*). Some states are never treated, but most states are treated multiple times. We assume that the effect of an extra $2,000 per year (the returns from a $1 minimum wage increase for a full-time worker) is roughly constant across minimum wage earners earning slightly different amounts. This is required for our selection of analysis window (there were certainly minimum wage increases before 2003) and our econometric structure which treats every cent of premium as equivalent, regardless of base or timing. Provided this assumption holds, our approach adheres to the recommendations of Goodman-Bacon (2018) and Baker et al. (2021) and our treatment estimates are unbiased.

However, we are not immune from the critiques made by Wolfers (2006), and with multiple treatments in close succession, we are unable to identify a new long-run equilibrium because states are regularly getting new treatments by way of increases in the minimum wage. This poses two risks to our analysis. First, we cannot say with certainty whether the reductions in marriage and divorce are temporary (merely a delay) or permanent. Regardless, the demography of marriage and divorce is shifted, and the importance of these findings remain. Second, states that adopt one minimum wage increase are likely to adopt another. Without knowing the exact timeline for effects on marriage and divorce, it is *possible* that our observed reduction in divorce can be partly attributed to reductions in vulnerable marriages from a prior minimum wage change. An approach that was able to carefully examine the *quality* of marriages formed might be able to distinguish whether these missing divorces were from negatively selected marriages that never took place, or (more) positively selected marriages that survived with reduced stress.

Similarity of the ACS and CPS Results

To verify that the ACS and CPS results are actually identical, we combine the data sets together and estimated a pooled model, adding an indicator term for data set (operationalized as an indicator for the CPS). We run our main specification with the addition of interacting our variable of interest, the minimum wage premium, with this indicator. As shown in Table S7, all estimates are statistically insignificant, indicating that our results from the two data sets are statistically indistinguishable.

Falsification Tests

It is possible that there is enough variability in state family formation trends for our estimated result to be a consequence of noise or state-specific time-varying factors independent of minimum wage changes. In order to determine the likelihood of this possibility, we conduct a series of falsification tests in which we estimate our preferred model for a placebo treatment.

In Figure S1, we present kernel density plots of the beta coefficients from our placebo treatment tests. To conduct these tests, we randomly assigned a state’s entire minimum wage history to another state (but keeping the recipient state’s original family formation history), sampling with replacement until all states had a randomized treatment. For example, we may have matched Connecticut’s minimum wage history with Texas’s family formation history. We then estimated the beta coefficient on the minimum wage premium. We repeated this process 1,000 times for each group (2x – men and women), data set (2x – ACS and CPS), and outcome (2x – marriage and divorce. For ease of comparison, we plot the estimated coefficients for men and women together on one kernel density plot. If our estimates are driven by true variation in a state’s own minimum wage history, and not by broader economic conditions, spurious covariance, or other unmodeled factors, we would expect to estimate a coefficient of 0, indicating no effect from the false minimum wage history.

For convenience we plot the $\hat{\beta}=0$ line. First, we note that all of the estimates are effectively zero. Not only are the parameter estimates extremely close to zero, but none of the estimates were statistically significant in any of the 1,000 x 6 regressions we estimated (t-statistics ranged from .09 to .34 for the CPS data, and .10 to .26 for the ACS). Secondly, while there appear to be small differences between men and women, none of these differences are substantively important – they are all slightly different flavors of zero.

These results present strong evidence against an unobserved coincident effect driving our results. For example, if marriages and divorces were associated with another phenomenon driving our results (e.g., unobserved religiosity), then we would expect to see some proportion of the minimum wage effects in our placebo results since that underlying and unobserved effect would still be exerting influence. Of course, it is possible that minimum wages and unobserved religiosity covary – but if that is the case, then there is no way to identify the independent effects. We think this is highly unlikely across 50 states and Washington, D.C. over the course of 13 years.

References

Baker, A., Larcker, D. F. & Wang, C. C. Y. (2021). How much should we trust staggered difference‐in‐differences estimates?. https://ssrn.com/abstract=3794018 or <https://doi.org/10.2139/ssrn.3794018>

Goodman‐Bacon, A (2018). Difference‐in‐differences with variation in treatment timing (NBER Working Paper 25018). <http://www.nber.org/papers/w25018>

UC Berkeley Labor Center (2015). Low‐Wage California: 2014 CHARTBOOK. <https://laborcenter.berkeley.edu/pdf/2014/chartbook-data-and-methods.pdf>

Wolfers, J. (2006). Did unilateral divorce laws raise divorce rates? A reconciliation and new results. American Economic Review, 96(5), 1802–1820. https://doi.org/10.1257/aer.96.5.1802

Table S1. Minimum wage premia and changes in minimum wages 2003-2014

|  |  |
| --- | --- |
| **Minimum wage premium (State - Federal)** |  |
| Average among all states | $0.38 |
| Average among states with state minimum wage above federal | $0.96 |
| **Between-year within-state differences (Current - Previous)** |  |
| Average among states with any increase (inc. Federal) | $0.54 |
| Average among states with state-initiated increase | $0.50 |
| **Number of changes over analysis period (Current** $\boldsymbol{\neq}$ **Previous)** |  |
| Average number of changes (inc. Federal) | 5.51 |
| Average number of state-initiated changes | 3.04 |

Notes: Authors’ analysis of state-level minimum wage changes. Main analysis covers reference years 2004 to 2015, but minimum wage variables are lagged at least one year, covering 2003 to 2014.

Table S2. Minimum Wage Increases Raise Earnings

| Min. wage | Males 18-35, CPS | Females 18-35, CPS | Males 18-35, ACS | Females 18-35, ACS |
| --- | --- | --- | --- | --- |
| Same year | 0.0642*** | 0.0722*** | 0.0431*** | 0.0585*** |
|  | (0.0114) | (0.0117) | (0.0110) | (0.0105) |
| One year lag | 0.0567*** | 0.0726*** | 0.0499*** | 0.0609*** |
|  | (0.0122) | (0.0117) | (0.00891) | (0.00957) |
| Two year lag | 0.0266** | 0.0388*** | 0.0371*** | 0.0475*** |
|  | (0.0117) | (0.0129) | (0.00940) | (0.00983) |

Notes: All regression results control for US-wide recession indicator, increases in the Federal minimum wage, logarithm of state-level GDP, state-specific pre- and post-trends. The population analyzed are households where at least one worker earns less than $20 per hour. For the CPS sample the earnings measure is log of household income, for the ACS log of family income.

*** indicates statistically significant at ($\alpha<0.01$); ** indicates statistically significant at ($\alpha<0.05$)

Table S3. Earnings Increase is Not Attributable to Change in Hours Worked

| Min. wage | Males 18-35, CPS | Females 18-35, CPS | Males 18-35, ACS | Females 18-35, ACS |
| --- | --- | --- | --- | --- |
| Same year | -0.113 | 0.134 | 1.853 | -1.836 |
|  | (0.121) | (0.112) | (6.373) | (5.265) |
| One year lag | -0.154 | 0.00227 | -4.608 | -2.069 |
|  | (0.125) | (0.114) | (6.146) | (5.010) |
| Two year lag | -0.306** | -0.178 | -12.38* | 0.845 |
|  | (0.155) | (0.119) | (6.521) | (6.057) |

Notes: All regression results control for US-wide recession indicator, increases in the Federal minimum wage, logarithm of state-level GDP, state-specific pre- and post-trends. The population analyzed are households where at least one worker earns less than $20 per hour. Dependent variable is usual hours worked per week.

** indicates statistically significant at $\alpha<0.05$

* indicates statistically significant at $\alpha<0.1$

Table S4. Changing Population Definition Does Not Alter Results: One-year minimum wage lag

|  | Males 18-35, CPS | Females 18-35, CPS | Males 18-35, ACS | Females 18-35, ACS |
| --- | --- | --- | --- | --- |
|  | Marriage | | | |
| <$20/hour (baseline) | -0.0204*** | -0.0226*** | -0.0210*** | -0.0214*** |
|  | (0.00553) | (0.00640) | (0.00399) | (0.00454) |
| <200% FPL | -0.0126* | -0.0220*** | -0.0230*** | -0.0219*** |
|  | (0.00765) | (0.00742) | (0.00448) | (0.00522) |
| <$16/hour | -0.0184*** | -0.0196*** | -0.0165*** | -0.0191*** |
|  | (0.00589) | (0.00665) | (0.00410) | (0.00457) |
|  | Divorce | | | |
| <$20/hour (baseline) | -0.00523*** | -0.000630 | -0.00354*** | -0.00396*** |
|  | (0.00167) | (0.00193) | (0.00112) | (0.00128) |
| <200% FPL | -0.00769*** | -0.00636** | -0.00506*** | -0.00696*** |
|  | (0.00261) | (0.00272) | (0.00160) | (0.00182) |
| <$16/hour | -0.00501*** | -0.00139 | -0.00287** | -0.00403*** |
|  | (0.00186) | (0.00208) | (0.00112) | (0.00138) |

Note: Estimates are for a one year minimum wage lag on the outcome of interest. Each point estimate is from a separate regression that includes the following controls: US-wide recession indicator, increases in the Federal minimum wage, logarithm of state-level GDP, state-specific pre- and post-trends.

* $\alpha<0.10$ **$\alpha<0.05$ *** $\alpha<0.01$

Table S5. Pooling Genders Does Not Alter Results: $20/hour sample definition

|  | One year lag | | Two year lag | |
| --- | --- | --- | --- | --- |
|  | Adults 18-35, CPS | Adults 18-35, ACS | Adults 18-35, CPS | Adults 18-35, ACS |
|  | Marriage | | | |
| Minimum wage premium | -0.0216*** | -0.0211*** | -0.0201*** | -0.0246*** |
|  | (0.00555) | (0.00407) | (0.00529) | (0.00396) |
|  | Divorce | | | |
| Minimum wage premium | -0.00296** | -0.00372*** | -0.00201 | -0.00416*** |
|  | (0.00148) | (0.00103) | (0.00153) | (0.00107) |

Note: Estimates are for a one- or two-year minimum wage lag on the outcome of interest. Each point estimate is from a separate regression that includes the following controls: US-wide recession indicator, increases in the Federal minimum wage, logarithm of state-level GDP, state-specific pre- and post-trends.

* $\alpha<0.10$ **$\alpha<0.05$ *** $\alpha<0.01$

Table S6. Results are Robust to Alternate Specifications: ACS data; $20/hour sample definition; one-year lag of minimum wage

|  | (1) | (2) | (3) | (4) | (5) | (6) |  |
| --- | --- | --- | --- | --- | --- | --- | --- |
|  | Marriage | | | | | |  |
| Women | -0.0605*** | -0.0599*** | -0.0596*** | -0.0586*** | -0.0482*** | -0.0214*** |  |
| Men | -0.0220** | -0.0228** | -0.0268*** | -0.0242** | -0.0470*** | -0.0210*** |  |
|  | Divorce | | | | | |  |
| Women | -0.00189 | -0.00209 | -0.00297 | -0.00226 | -0.00741*** | -0.00396*** |  |
| Men | -0.0132*** | -0.0129*** | -0.0124*** | -0.0123*** | -0.00631*** | -0.00354*** |  |
|  |  |  |  |  |  |  |  |
| Detrend | X | X | X | X | X | X |  |
| Fed MinWage |  | X | X | X | X | X |  |
| Recession |  |  | X | X | X | X |  |
| State GDP |  |  |  | X | X | X |  |
| Pooled Time Trend |  |  |  |  | X |  |  |
| State Time Trend |  |  |  |  |  | X |  |

Note: Estimates are for a one-year minimum wage lag on the outcome of interest. Each point estimate is from a separate regression that includes adds additional controls moving from (1) to (6). (1) detrended pre-treatment, (2) increases in the Federal minimum wage, (3) US-wide recession indicator, (4) logarithm of state-level GDP, (5) pooled time trend, and (6) state-specific post-trends.

* $\alpha<0.10$ **$\alpha<0.05$ *** $\alpha<0.01$

Table S7. ACS and CPS Results are Statistically Indistinguishable

|  | Males 18-35 | Females 18-35 |
| --- | --- | --- |
|  | Marriage | |
| CPS x Minimum wage premium | -0.00530 | -0.000700 |
|  | (0.00653) | (0.00732) |
|  | Divorce | |
| CPS x Minimum wage premium | -0.000725 | 0.000673 |
|  | (0.00147) | (0.00176) |

Note: CPS and ACS data are combined; estimates are the interaction between an indicator for CPS data and a one-year lag minimum wage premium on the outcome of interest. Each variable is also included in a non-interacted form. Each point estimate is from a separate regression that includes the following controls: US-wide recession indicator, increases in the Federal minimum wage, logarithm of state-level GDP, state-specific pre- and post-trends. The population analyzed are households where at least one worker earns less than $20 per hour. All estimates are statistically insignificant (p>0.10).

Figure S1. Falsification Tests: Distribution of $\beta$ from Randomly Assigned Treatment

(Population: $20/hour definition, Minimum Wage: one-year lag)

First marriage

Panel A Panel B

**
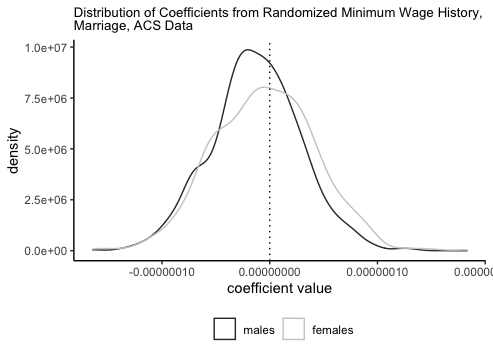

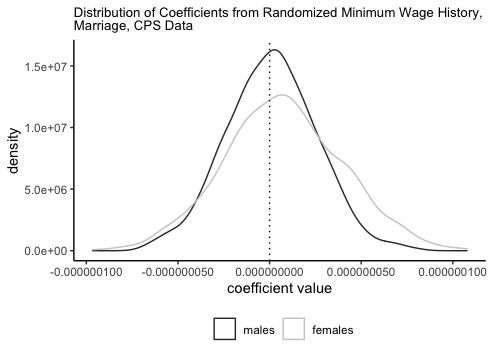
**

Divorce


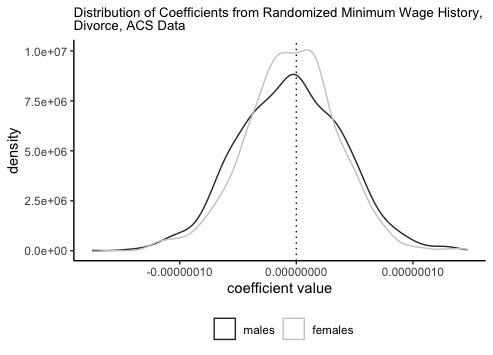
 Panel C Panel D


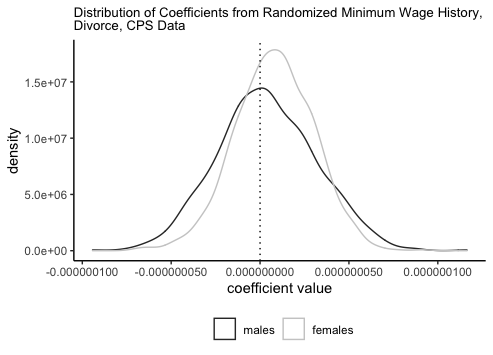


1. The 2019 version of the ACS has re-established the question on exact weeks worked (last used in 2007). [↑](#footnote-ref-1)
2. <http://www.economics.uci.edu/~dneumark/MW_LW%20dataset%20updated%20through%202019%20-%201-01-20%20Update.csv> [↑](#footnote-ref-2)
3. <https://equitablegrowth.org/wp-content/uploads/2016/09/090716-WP-Historical-min-wage-data.pdf> [↑](#footnote-ref-3)
